# Chinese Herbal Medicine as a Potential Treatment of Abdominal Aortic Aneurysm

**DOI:** 10.3389/fcvm.2018.00033

**Published:** 2018-04-20

**Authors:** Sai Wang Seto, Dennis Chang, Hosen Kiat, Ning Wang, Alan Bensoussan

**Affiliations:** ^1^NICM Health Research Institute, Western Sydney University, Penrith, Australia; ^2^Faculty of Medicine, University of New South Wales, Sydney, Australia; ^3^School of Medicine, Western Sydney University, Penrith, Australia; ^4^Faculty of Medicine and Health Sciences, Macquarie University, Sydney, Australia; ^5^Key Laboratory of Xin’an Medicine, Ministry of Education, Hefei, China; ^6^College of Pharmacy, Anhui University of Chinese Medicine, Hefei, China; ^7^Institute for Pharmacodynamics and Safety Evaluation of Chinese Medicine, Anhui University of Chinese Medicine, Hefei, China

**Keywords:** Chinese herbal medicine, abdominal aortic aneurysm, vascular biology and disease, atherosclerosis, inflammation mediators, oxidative stress

## Abstract

Abdominal aortic aneurysm (AAA) is an irreversible condition where the abdominal aorta is dilated leading to potentially fatal consequence of aortic rupture. Multiple mechanisms are involved in the development and progression of AAA, including chronic inflammation, oxidative stress, vascular smooth muscle (VSMC) apoptosis, immune cell infiltration and extracellular matrix (ECM) degradation. Currently surgical therapies, including minimally invasive endovascular aneurysm repair (EVAR), are the only viable interventions for AAAs. However, these treatments are not appropriate for the majority of AAAs, which measure <50 mm. Substantial effort has been invested to identify and develop pharmaceutical treatments such as statins and doxycycline for this potentially lethal condition but these interventions failed to offer a cure or to retard the progression of AAA. Chinese herbal medicine (CHM) has been used for the management of cardiovascular diseases for thousands of years in China and other Asian countries. The unique multi-component and multi-target property of CHMs makes it a potentially ideal therapy for multifactorial diseases such as AAA. In this review, we review the current scientific evidence to support the use of CHMs for the treatment of AAA. Mechanisms of action underlying the effects of CHMs on AAA are also discussed.

## Introduction

Abdominal aortic aneurysm (AAA) is an irreversible condition where the abdominal aorta is dilated with a diameter of at least 30 mm in an adult. The risk factors include male gender, age >75 years, pervious vascular disease, hypertension, smoking, family history and hypercholesterolemia (1). AAAs are often asymptomatic and remain undiagnosed until the time of rupture leading to surgical emergency with a high mortality rate of 50–80% (2).  Numerous clinical studies have demonstrated that the risk of AAA rupture markedly increased and surgical repair is required when the maximum diameter reaches 50–55 mm (3, 4). So far, only surgical therapies are available for AAAs but increasing number of reports have suggested that this “50 mm diameter criterion” does not fully reflect the severity of AAA in patients completely, limiting the effectiveness of the surgical therapy for majority of AAAs which measure <50 mm.

Traditionally, AAA was considered as a simple biomechanical problem due to an irreversible structural change on the aortic wall. However, it has been now been widely accepted that the changes in the biomechanical environment can trigger multiple pathological processes involving morphological, cellular and biochemical changes in the vasculatures of aorta (5, 6). Although the exact mechanisms are not clear, the major pathophysiological mechanisms of AAA involve inflammation, oxidative stress, proteolysis and apoptosis (7–10). These are characterized by degradation of elastin fibres, increased collagen expression, excessive immune cells infiltration, vascular smooth muscle cell (VSMC) apoptosis and excessive medial neovascularisation in the AAA segments (8, 11, 12). A large number of studies have been conducted to assess the efficacy of potential drug therapies including angiotensin converting enzyme (ACE) inhibitor, calcium channel blocker, lipid lowering agents, anti-coagulants and anti-inflammatory agents for reducing AAA expansion rate. Although the initial studies in animal models demonstrated promising effects of these agents in suppressing AAA development and progression in animal models, the randomised clinical trials in human subjects with AAA failed to replicate these findings. To date, no viable drug treatment is available to diminish AAA expansion; current management of AAA largely relies on reduction of cardiovascular factors. Therefore, alternative and novel therapeutic interventions that can slow down AAA progression should be investigated.

Recent proteomic, signalling and metabolomics data have clearly demonstrated the complex interactions between a network of signalling molecules and cells in AAA. Thus, the current “one drug one target” strategy may not be ideally suited to address the different facets of this complex disease effectively. Indeed, some contemporary treatment strategies which focus on the use of multiple compounds/component drugs (e.g., Polypill) (13, 14) to overcome the limitations of single target therapy have gained popularity for the management of these complex diseases.

Traditional Chinese medicine (TCM), including Chinese herbal medicines (CHM) and acupuncture, with its unique theory and long history, has been used for the management of cardiovascular diseases for thousands of years in China. In recent years, there has been an increasing recognition of the health benefits of CHM, which are supported mounting evidence from pre-clinical and clinical studies (15–17). Traditionally, CHM is prescribed in the form of mixtures of plant, animals or minerals and often contains numerous bioactive components. Accumulated evidence has suggested that the multi-component and multi-target property of CHM may help address diseases that have multifactorial/multisystem pathophysiological components (e.g., AAA) (18–20).

In the current paper, we aim to provide an overview of major pathologies of AAA and to review current scientific evidence of the use of CHM highlighting its potential as a novel treatment strategy for AAA. We conducted a literature review on MEDLINE, SCOPUS, Web of Science and Cochrane Library database. The following key words were used either as single or combined searches: “abdominal aortic aneurysm”, “traditional Chinese medicine”, “herbs”, “natural product” and “herbal formulation”.

## Current Treatment Options and Limitations

Surgical/mechanical intervention is the only effective treatment to prevent AAA rupture and aneurysm-relate death for large AAA ≥55 mm (21). Despite the relatively low failure rate (~0.3%) and long term durability of the graft in most patients that survived from the elective open surgical repair (22, 23), this complex intervention has a 30 day mortality rate of 5% and the operative mortality increases markedly in medically unfit patients (24, 25). Over the last two decades, minimally invasive endovascular aneurysm repair (EVAR) has gained acceptance as an alternative to open surgical repair (25). Although EVAR has been shown to have clear benefit over conventional open surgical repair, it is associated with a number of issues such as endoleak and graft migration (26). Even though advancement in surgical technique and EVAR have greatly improved the treatment outcomes in patients with large AAA, a large US screening study showed that 90% of AAAs identified were small AAA (<55 mm) (27) where the benefit in reducing AAA rupture is largely outweighed by the risk of surgical intervention (28). Many countries, such as the United Kingdom, provide all men over the age of 65 a one-off non-invasive AAA screening with the aim to reduce AAA-related mortality through early detection of this fatal condition. Majority of patients identified with AAA do not require immediate surgical intervention (29) which dramatically increased the pool of small AAA patients seeking pharmacological treatments to slow down the AAA growth.

Over the last two decades, based on a better understanding of the pathogenesis of AAA, a large number of pre-clinical studies have highlighted a number of potential pharmacological therapies for limiting AAA growth in small animal models and *ex vivo* experiments of human AAA biopsy (30–35). However, translating of these findings into effective treatments has proven challenging with the reports from major clinical trials (36–38) that have all shown negative results. There are a number of possible reasons accounting for this failure. Firstly, although the current AAA animal models mimic many features of AAA, they do not accurately mirror the chronic pathological changes in human AAA. For instant, AAAs in human usually develop and progress over decades with the presence of an intraluminal thrombus (ILT), while aortic dilatation in animal models occurs within days or weeks without consistent presence of ILT (39). Secondly, most of the current studies using AAA models assessed the effects of interventions on the initiation of AAA rather than its progression. Doxycycline, a tetracycline analogue, has been demonstrated to prevent AAA formation *via* MMPs inhibition in a variety of animal studies (20–23). However, in a randomized, placebo-controlled, double-blind trial, 18 month treatment with, doxycycline failed to inhibit AAA progression and the need for AAA repair (38). These findings suggest that AAA initiation and formation is a different process from AAA progression, hence, more carefully designed animal studies, such as chronic and pre-established models, are needed to assess the effectiveness of an intervention in suppressing AAA growth rather than initiation. Thirdly, most of the human AAA samples were obtained from the surgical repair of advanced/large AAA. Studies using these samples only represent the advance stage of the disease where pharmacological therapies, which target the earlier stage of the diseases, are unlikely to be effective. Finally, it is worth pointing out that most current treatment strategies only target one specific mechanism (e.g., or. mast cell inhibition) of AAA. Given the fact that AAA involves a complex interplay between numerous cellular and molecular mechanisms, a multi-component and multi-target approach may be more appropriate in the treatment of AAA.

## CHM as Potential Drug Therapy for AAA

In CHM, multiple herbs are often combined as a complex mixture/formula for the treatment of various health conditions based on the principle of “Jun (emperor) - Chen (minister) - Zuo (assistant) - Shi (courier).” In complex formulations, individual components can act directly and/or synergistically on individual/multiple therapeutic targets. Some of these interactions may also lead to increased bioavailability or decreased toxicity (15, 40). This multi-component and multi-target approach may offer a better option for the management of diseases with complex pathophysiology, such as AAA (15, 17).

### Use of CHMs for AAA

 Vascular protective effects of single herbal extracts and/or their isolated bioactive component on AAA have been demonstrated in a number of animal studies (Table 1). However, direct evidence to support the use of complex herbal formulations for AAA is limited even though a number of complex Chinese herbal formulations, such as Si Miao Yong An (SMYA) (51), Sai Lou Tong (SLT) (16), Tong Xin Luo (TXL) capsule (52) and Huang Lian Jie Du Tang (53), have been shown to have vascular protective effects. In a head-to-head comparison study between TXL capsule (consisting of *Radix ginseng*, *Buthus martensii*, *Hirudo medicinalis*, *Eupolyphaga seu steleophaga*, *Scolopendra subspinipes*, *Periostracum cicadae*, *Paeoniae rubra*, *Semen ziziphi spinosae*, *Lignum dalbergiae odoriferae*, *Lignum santali albi*, and *Borneolum syntheticum*) and simvastatin, both TXL and simvastatin at high dose showed to enhance plaque stability with similar lipid-lowering, anti-inflammatory and anti-oxidative effects in a rabbit model of atherosclerosis. Interestingly, low-dose TXL was shown to exhibit similar anti-inflammatory effect to that of high-dose simvastatin, indicating that TXL possesses a greater anti-inflammatory potency compared to simvastatin (54). In a diet-induced atherosclerosis model in rabbits, SMYA [consisting of *Flos lonicerae japonicae* (Jinyinhua), *Radix scrophulariae ningpoensis* (Xuanshen), *Radix angelicae sinensis* (Danggui) and *Radix glycyrrhizae uralensis* (Gancao)], has been shown to reduce various pathologies of AAA. Oral administration of SMYA significantly lowered abdominal aortic MMP-9 and NF-κB expression and inflammatory cytokines, such as MCP-1, IL-1 and IL-5 systemically. In addition, SMYA promoted atherosclerotic plaque stability through reduction of macrophages infiltration and inhibition ECM degradation (51). Although the underlying effects and molecular mechanisms were not investigated using an AAA specific animal model, findings from this study has clearly demonstrated the potential beneficial effects of SMYA for AAA.

**Table 1:** Summary of the effects of Chinese herbs and herbal bioactive ingredients on AAA in animal models.

**Herb Extract/Bioactive ingredient**	**Model of Study**	**Proposed Mechanisms**	**Ref**
Ginsenosides Rb1	ApoE^−/−^ mouse AngII-induced AAA	↓MMP production↓inflammatory cell infiltration↓ECM degradation↓ JNK and p38 MAPK activation	(41)
Monascus purpureus(Chinese Red Yeast Rice)	ApoE^−/−^ mouse AngII-induced AAA	↓Serum cholesterol↓ICAM1 ↓VCAM1↓MMP-2 and -9 expression↑Macrophage migration inhibitory factor	(42)
Quercetin	C57/BL6 mouse extraluminal calcium chloride-induced AAA	↓superoxide dismutase activities ↓glutathione peroxidase expression↓JNK signalling↓MMP-2 and -9 activity	(43)
Epigallocatechin-3-gallate (Green tea polyphenol)	Rat extraluminal calcium chloride-induced AAA	↓Inflammatory cytokine expression↓MMP-9 expression and activity↑Tissue inhibitors of MMP-1	(44)
Resveratrol	ApoE^−/−^Ace2^−/y^ mouse AngII-induced AAA	↑serum and aortic ACE2 activity↓Sirtuin 1 activity↓MMP-2 and -9 expression↓Angiotensin type-1 receptor↓Akt and ERK signalling	(33)
Salvianolic acid C	ApoE^−/−^ mouse AngII-induced AAA	↓MMP-2 and -9 activity↓Elastin fragmentation↓Macrophage infiltration	(45)
Salvianolic acid A	ApoE^−/−^ mouse AngII-induced AAA	↓MMP-2 and -9 activity↓Elastin fragmentation↓Macrophage infiltration	(46)
Baicalein	ApoE^−/−^ mouse AngII-induced AAA	↓MMP-2 and -9 activity↓Angiotensin type 1 receptor↓MAPK signalling↓Inflammatory cell infiltration↓Reactive oxygen species (ROS) production	(47)
Ginkgo biloba extract (EGb 761)	C57/BL6 mouse extraluminal calcium chloride-induced AAA	↓Elastin fragmentation↓MMP-2 and -9 activity↓ROS production↓NFκB signalling	(48)
Dietary phytoestrogens	C57/BL6 mouse intraluminal elastase-induced AAA	↓Inflammatory cell infiltration↓MMP-9 activity↓Inflammatory cytokines level	(49)
Curcumin	ApoE^−/−^ mouse AngII-induced AAA	↑SOD expression↓ERK1/2 signalling↓Inflammatory cytokines level↓Macrophage infiltration	(50)

MMP, Matrix metalloproteinases; JNK, c-Jun N-terminal kinases; ICAM1, Intercellular Adhesion Molecule 1; VCAM1, Vascular Adhesion Molecule 1; ERK, Extracellular signal–regulated kinases; NFκB, Nuclear Factor Kappa-Light-Chain-Enhancer of Activated B Cell; SOD, Superoxide Dismutase.

A recent study by Xiong et al (55) in ApoE^−/−^ mouse showed that Shexiang Tongxin dropping pill (STDP), a seven herbs formulation consisting *Radix rhizoma ginseng*, *Calculus bovis*,* bear gall*, *Venenum bufonis*, *borneol, moschus*, and *Salvia miltiorrhiza*, produced its anti-atherosclerotic effects *via* multiple mechanisms including reduced pro-inflammation cytokines (IL-2, Ilt-6, TNF-α and γ-IFN), upregulated serum anti-oxidative enzyme levels (superoxide dismutase (SOD) and glutathione), decreased aortic expression of miR-21a, miR-126a, miR-155, and increased miR-20a expression (55). It is worth noting that differential expression of various miRNAs has been demonstrated in aneurysmal aortic samples which is strongly associated with the development of AAA. For example, miR155 has been shown to play a vital role in vascular inflammation via programming of macrophages (56). In line with that, a later study has demonstrated that miR155 is overexpressed in both the aneurysmal site and serum of AAA patient (57). Moreover, both miR-21a and miR-126, which act as key regulators of vascular remodelling by controlling VSMC apoptosis and vascular inflammation, have been shown to be upregulated in abdominal aortic aneurysmal tissues and (58, 59). In another study, Xiaoxianggou, consisting of *Ficus pandurata hance var. angustifolia Cheng*, *Ficus panduram Hane var.hoiophylla Migo* and *Ficus erecta Thunb.var. bcecheyana (Hook.et Am.) King*., has been shown to reduce atherosclerotic lesion formation via downregulation of miR-203 expression in an endogenous high Ang II ApoE^−/−^ mouse model (60). miR-203 has been shown to promote VSMC dysfunction and aortic stiffness in aged mouse (61). Therefore, modulation of miRNA expressions by CHMs could represent a novel AAA management strategy.

Western medicine is characterized by its rapid effect in controlling and relieving of symptoms, unfortunately the adverse effects associated with some of the Western or synthetic medicines have limited their applications (62–65). It has been reported that CHMs when used in conjunction with Western medicine can produce greater therapeutic effects and/or less adverse effects than Western medicine alone (66, 67). Didang decoction (DDD), a traditional Chinese formula (consisting of *Rheum rhabarbarum*, *Hirudo medicinalis*, *Prunus persica* and *Tabanus atratus*), has been shown to produce a greater effect on attenuating vascular lesion and endothelial injury than metformin in a diabetic model in rats (68). Jiang et al showed that artesunate, a derivate of artemisinin from sweet wormwood, attenuated formation of atherosclerosis lesion via reduction of vascular inflammation in high-fat fed ApoE^−/−^ mice. When combined with rosuvastatin, a lipid-lowering agent, artesunate produced a greater attenuation of atherosclerosis compared to artesunate and rosuvastain alone (69).

A recent systematic review and meta-analysis has reported that a combined therapy of CHMs and warfarin is more effective in preventing total thromboembolic events in patients with atrial fibrillation (70). Zhibitai, is a Chinese herbal comprised of a family of naturally occurring statins to treat blood lipid disease. When combined with low dose of atorvastatin (10 mg), Zhibitai has been shown to improve blood lipid profile and reduce inflammation, similar to that of higher doses of atorvastatin (20 and 40 mg) with less adverse effects in patients with coronary heart disease (67). Given the role of hyperlipidemia and inflammation in AAA development and growth, the results of this study suggest that the combination therapy between CHM and Western medicine may offer additional benefits.

### Mechanisms of Action

It is now generally accepted that inflammation, oxidative stress and extracellular matrix (ECM) degradation are the three fundamental factors contributing to the development of AAA (8, 71–73). In this section, the mechanisms of action underlying the effects of CHM against these major pathophysiological processes of AAA are discussed.

#### Anti-Inflammation

Chronic inflammation plays a significant role in the development and progression of AAA. Although little is known about the initiation of AAA development, histological analysis has revealed a transmural degenerative process in the aortic wall involving a number of inflammatory cells, such as macrophages, monocytes and lymphocytes (31, 35, 74). In addition to dense inflammatory cells infiltration within the outer media and adventitia of the aortic wall, multiple pro-inflammatory cytokines and mediators have also been identified in the aortic aneurysm as well as in the circulation (32, 72, 75). The enhanced pro-inflammatory cytokines levels promote activation of immune cells and stimulate production of matrix metalloproteinase (MMP) at the aneurysmal site, directly contributing to the degradation of major extracellular structural proteins such as elastin and collagen. Although whether inflammation and MMP activation is the direct cause of AAA remains to be determined, the critical role of inflammation and immunity changes in AAA is well accepted. Indeed, a large body of evidence have demonstrated that anti-inflammatory or immune-based intervention may be an effective therapeutic and preventive approach for AAA. For example, doxycycline, a tetracycline analogue, has been demonstrated to prevent AAA formation *via* MMPs inhibition in a variety of animal studies (76–79) and to limit AAA growth in two small clinical trials (7, 80). Similarly, hydroxymethylglutaryl-coenzyme A (HMG CoA) reductase inhibitors (statin) has also been suggested to benefit AAA through its ability to inhibit several MMPs and immune cells activity and downregulation of various inflammation signalling molecules expression as demonstrated in a number of *in vitro* and *in vivo* studies (81–84). Although doxycycline and statins failed to limit AAA growth in clinical trials (21, 85, 86), the importance of anti-inflammation strategies in AAA management should not be ignored. Instead, more specific MMP inhibitors with better pharmacokinetics and tissue absorption should be developed.

A large number of CHMs possess anti-inflammatory properties, some of which have demonstrated beneficial effects in the treatment and prevention of various cardiovascular conditions. For example, *Aralia cordata* (Tu Dan Gui), have been shown to have significant anti-inflammatory effects. Ent-pimara-8(14), 15-dien,19-oic acid (pimaradienoic acid, PA) is believed to be responsible for this effect through reduction of the nitric oxide (NO), prostaglandin E2 (PGE2) and interleukin-6 (IL-6) generation and inducible NO synthase (iNOS) and cyclooxygenase-2 (COX-2) expression via inactivation of MAPK signalling following IκB degradation and NF-κB activation in cultured macrophages (87). Similarly, kaurenoic acid (KA), a major diterpenoid from the root of *Aralia continentalis* (Dong Bei Tu Dan Gui), displayed anti-inflammatory effects in both *in vitro* and *in vivo* models via inhibition of iNOS and COX-2 expression (88). A later study by the same group showed that the anti-inflammatory effects of KA is mediated *via* activation of the nuclear factor erythroid 2-related factor 2 (Nrf2) (89). Considering the demonstrated role of COX-2, iNOS, NF-κB and Nrf2 signalling in the progression and development of AAA (90–92), these studies have highlighted the potential of CHMs in control of various inflammatory processes during AAA and its related vascular conditions, such as atherosclerosis.

####  Anti-*Oxidative Stress*

Reactive oxygen species (ROS) are a small group of chemically reactive molecules which are involved in vascular functions such as smooth muscle contraction, angiogenesis and endothelial functions (93). However, excessive ROS generation due to dysfunction of either enzymic and non-enzymic mechanisms [such as enhanced activation of xanthine oxidase (XO), lipoxygenase (LOX) and NADPH oxidase (NOX), overexpression of iNOS and impaired mitochondrial respiratory electron transport chain (ETC)] is closely associated with a number of vascular diseases, including AAA (94, 95). Indeed, increased oxidative stress and ROS over generation has been shown to play a pivotal role in AAA progression and development (71, 96). For instant, both *in vitro* and *in vivo* studies have demonstrated that overproduction of ROS exacerbates various AAA pathogenesis including vascular smooth muscle cell (VSMC) migration, metalloproteinases (MMP) activation, extracellular matrix (ECM) degradation and endothelium dysfunction (92, 97–100). In human studies, high-grade oxidative stress has been observed in AAA patients, suggesting the possible protective effect of anti-oxidant supplement for AAA (101, 102). Xiong et al showed that inhibition of NADPH oxidase by apocynin significantly suppressed AAA formation in a mouse model of AAA (103). Later study showed that angiotensin II-induced AAA is exacerbated in NADPH oxidase deficiency mice (104). These studies have clearly demonstrated that ROS is an important target to reduce AAA progression (103, 105, 106).

Many CHMs have been shown to have remarkable anti-oxidative properties (107, 108). For instance, Sailuotong (SLT), a standardised three-herb formula combining specific dosages of *Panax ginseng* (Ren Shen), *Ginkgo biloba* (Yin Xin Ye), and *Crocus sativus* (Zhuang Hong Hua), has been shown to protect endothelial cell from ROS-induced apoptosis *via* enhancement of superoxide dismutase (SOD) activity and suppression of caspase-3 activation (16). EGb 761, a standardized extract of *Ginkgo biloba* is a well-known anti-oxidant (109, 110). Interestingly, several studies have shown that extract of *Ginkgo biloba* significantly reduced MMP production. In line with this, Wang et al (48) showed that EGb 761 limited AAA development in a mouse model possibly through reduction of oxidative stress and aortic MMP-2 and -9 activities (48). Resveratrol, a plant-derived polyphenolic compound, has been shown to prevent the formation of AAA induced by calcium chloride in mouse via multiple mechanisms, including anti-oxdiation and anti-inflammation (111). In a recent study, baicalein (BAI), the major bioactive component of *Radix Scutellariae Baicalensis* (Huang Qin), decreased the incidence of AAA in angiotensin II (AngII)-induced AAA in ApoE^−/−^ mouse. Moreover, BAI treatment significantly reduced AngII-induced aortic ROS production and MMP-2 and -9 activation (47). It is important to point out that, while these preclinical studies have clearly demonstrated anti-oxidative properties of CHMs, their clinical effects in AAA are yet to be determined.

#### Anti-ECM Degradation

The development of AAA is considered as a multi-stage process consisting of the initial loss of elastin mediated by VSMCs and collagen deposition followed by the production of ECM fragments and matrix destruction as a consequence of inflammation and eventually rupture (112, 113). It is generally accepted that cathepsins and matrix metalloproteinases (MMPs) are the two major proteases responsible for the ECM degradation (114, 115). High level of MMPs, including MMP-8, -9, -12 and 19, have been detected in advanced surgically removed aneurysm (116–119). In animal studies, knockout of MMP-2 and -9 reduced aneurysm formation (120, 121). Moran et al (122) have demonstrated that osteoprotegerin (OPG) is positively associated with AAA in human (122) and knockout of OPG limited AAA progression and reduced aortic MMP-2 and -9 expression in AngII-infused ApoE^−/−^ mouse (34). Similarly, deficiency of cathepsin K or cathepsin G attenuated AAA formation in multiple rodent models of AAA (123, 124). A large number of studies have been conducted to identify potential pharmaceutical candidates (e.g., MMP inhibitor) to inhibit ECM degradation. Unfortunately, most of these studies failed to lead to viable interventions despite promising results were obtained from animal studies or small scale clinical trials.

Several studies have demonstrated inhibitory effects of natural products on MMP. Abraham et al (2005) showed that Triphala, a traditional herbal concoction consisting of *Emblica officinalis*, *Terminalia bellirica* and *Terminalia chebul*a, has strong inhibitory activity on MMPs using gelatin zymography (125). In a similar study, *Azadirachta indica* (Neem) and *Aloe vera* have been shown to inhibit MMP-2 and -9 activity (126). In human aortic VSMC, carnosic acid, a phenolic compound found in various herbs such as *Rosmarinus officinalis* and *Salvia officinalis*, has been shown to inhibit MMP-9 expression through down-regulation of NF-κB (127). Studies have shown that activation of *c-jun* N-terminal kinase is a major mechanism of MMPs activation (128). Pimaric acid, a purified compound from *Aralia cordata*, has been shown to inhibit MMP-9 production via inhibition of *c-jun* activation in human aortic VSMC (129). In animal study, quercetin, a natural flavonoid found in many Chinese herbs, have been shown to limit AAA development and reduce aortic expression of MMP-2, -9, cathepsin B and cathepsin K in the calcium chloride-induced AAA model. Additionally, quercetin also increased tissue inhibitors of metalloproteinases (TIMP)−1 gene expression (130). A later study from the same group showed that the quercetin-mediated protective effects is regulated via the JNK/AP-1 signalling pathway (43). These studies clearly demonstrated the potential benefits of herbal medicine in the treatment of AAA and atherosclerosis *via* modulation of MMPs activity and their associated signalling pathways.

## Conclusion

 AAA is a multifactorial disease caused by oxidative stress and vascular inflammation ultimately leading to the destruction of the ECM structural proteins. Despite substantial efforts invested in the development of pharmaceutical interventions for AAA, little success has been achieved. Recent evidence clearly demonstrated the beneficial effects of CHMs against various major pathologies of AAA. Large number of single herb extract and their isolated active components have been studied in detail using various well-established animal model of AAA, including AngII-induced and calcium chloride-induced AAA mouse models. In contrast, the support of the potential of complex herbal formulations of AAA is largely based on animal model of atherosclerosis. It is important to point out that although atherosclerosis and AAA share many predisposing risk factors and exhibit similar immune and inflammatory cell infiltration at the lesion site, the underlying mechanisms mediating the development and progression are not always the same between these two diseases. Similarly, although rodent model of AAA been used extensively to understand the pathophysiology and evaluate potential treatment for AAA, these models of AAA do not fully recapitulate the condition in human. Therefore, the result observed from these animal studies should be interpreted with caution.

Similar to many Western therapies that have shown promising pre-clinical data, early evidence exists from pre-clinical studies demonstrating that CHMs (either as single herbs or as complex herbal formulations) also possess beneficial effects against AAA and/or other vascular pathologies via multiple mechanisms. These results highlight the advantage of multi-component and multi-target properties of CHM in the treatment for AAA. Robust randomized, controlled trials are needed to confirm and validate the clinical effectiveness of CHMs. It is important to point out that the translational potential of CHM approaches for AAA remains unknown and it is possible that CHMs would also fail to treat AAA in large, controlled trial given the complex nature of AAA and the stages at which patients can be identified. Moreover, combining Western medicine and CHM treatment may be a novel strategy to slow down AAA growth, this potentially important intervention should be fully explored with more well-designed clinical study. It will be especially important to test if combined treatment approach could be a valid option to control major AAA pathologies and AAA growth in patients who do not response to conventional therapy satisfactorily. Additionally, more research is needed to understand the interactions and synergistic effects among the multiple bioactive components of CHMs.

## Author Contributions

SS and DC conceived the study. AB, DC, HK and SS designed the study. SS searched all the relative papers and drafting this manuscript. DC, HK, NW and AB revised the manuscript. All authors read and approved the final manuscript.

## Conflict of Interest Statement

The authors declare that the research was conducted in the absence of any commercial or financial relationships that could be construed as a potential conflict of interest.
